# Evaluating *Salmonella pullorum* dissemination and shedding patterns and antibody production in infected chickens

**DOI:** 10.1186/s12917-022-03335-z

**Published:** 2022-06-24

**Authors:** Xuehuai Shen, Anyun Zhang, Ju Gu, Ruihong Zhao, Xiaocheng Pan, Yin Dai, Lei Yin, Qinghe Zhang, Xiaomiao Hu, Hongning Wang, Danjun Zhang

**Affiliations:** 1 Institute of Animal and Veterinary Science, Anhui Academy of Agricultural Sciences, Livestock and Poultry Epidemic Diseases Research Center of Anhui Province, Hefei, 230031 China; 2 Present address: Anhui Province Key Laboratory of Livestock and Poultry Product Safety Engineering, No.40 Nongkenan RD, Luyang District, Hefei, 230031 Anhui China; 3 Key Laboratory of Bio-Resource and Eco-Environment of Ministry of Education, Animal Disease Prevention and Food Safety Key Laboratory of Sichuan Province, College of Life Sciences, Sichuan University, Chengdu, 610064 China

**Keywords:** *Salmonella pullorum*, chickens, organ dissemination pattern, bacterial shedding, serum antibody, pullorum disease, horizontal transmission

## Abstract

**Background:**

Pullorum disease caused by *Salmonella pullorum* is one of the most important infectious diseases in the poultry industry, responsible for causing substantial economic losses globally*.* On farms, the traditional method to detect *S. pullorum* infection mainly involves the collection of feces and sera to test for antigens and antibodies, respectively, but the regularity of *Salmonella pullorum* dissemination in internal organs and shedding patterns and antibody production in infected chickens remains unclear. Herein we aimed to investigate the dissemination of *S. pullorum* to different organs and bacterial shedding patterns in the faeces as well as serum antibody production post-infection in chickens of different ages.

**Result:**

In this study, the liver and heart of 2-day-old chickens showed the highest copy numbers of *S. pullorum* at 6.4 × 10^6^ and 1.9 × 10^6^ copies of DNA target sequences/30 mg, respectively. In case of 10-day-old chickens, the percentage of *S. pullorum* fecal shedding (0%–40%) and antibody production (0%–56.6%) markedly fluctuated during the entire experiment; furthermore, in case of 42-week-old chickens, the percentage of birds showing *S. pullorum* shedding in the faeces showed a downward trend (from 63.33% to 6.6% in the oral inoculation group and from 43.3% to 10% in the intraperitoneal injection group), while that of birds showing serum antibody production remained at a high level (38.3% and 80% in the oral inoculation and intraperitoneal injection groups, respectively). We also performed cohabitation experiments, showed that 15% 10-day-old and 3.33% 42-week-old chickens were infected via the horizontal transmission in cohabitation with *S. pullorum* infected chickens, and revealed a high risk of horizontal transmission of *S. pullorum*.

**Conclusion:**

This study systematically evaluated the dissemination of *S. pullorum* in internal organs and bacterial fecal shedding patterns, and antibody production in infected chickens. Collectively, our findings indicate how to effectively screen *S. pullorum*-negative chickens on livestock farms and should also help in the development of measures to control and eradicate *S. pullorum*.

**Supplementary Information:**

The online version contains supplementary material available at 10.1186/s12917-022-03335-z.

## Background

*Salmonella pullorum* is highly adapted to fowl, in which it causes a widespread and devastating infection known as pullorum disease (PD, white diarrhea) [1]. PD is an acute systemic disease and associated with a high mortality rate; infected chickens show a range of symptoms, including anorexia, depression, diarrhea, and persistent cloacal infection [2]. Furthermore, although infected adult chickens may appear asymptomatic, *S. pullorum* can persist for many months in the spleen and reproductive tract, resulting in its vertical transmission to eggs and progeny [3]. All breeds of chickens of all ages are susceptible to *S. pullorum*, but infection mortality decreases with age, and many infected chickens develop latent and persistent infections [4]. The study found 20% of *S. pullorum* infected birds showed gradually decreasing bacterial numbers in the spleen and liver with clearance between 20-25 weeks of age, while in females the decline is interrupted by the onset of sexual maturity which leads to reduced T cell responsiveness [5]. *Salmonella* pathogenicity island (SPI), such as SPI-2 and SPI-19, were involved in mediating the inhibition of host immune responses, resulting in persistent colonization of *S. Pullorum* in hosts [3, 6].

Some studies have reported that the incidence of PD is no longer an issue in developed countries, but it still continues to persist in developing countries, with the detrimental impact of *S. pullorum* being substantially underestimated [7]. *S. pullorum* is an intracellular parasite, and although treatment with drugs is sufficient to manage clinical symptoms, complete elimination of bacteria is a challenge [2], which eventually leads to the development of subclinical persistent infections. After infection, *S. pullorum* can evidently modulate host immunity, with the antibody response persisting for >40 weeks [8]. Therefore, to completely eliminate *S. pullorum*, comprehensive prevention and control measures are highly desirable, in addition to the establishment of *Salmonella*-negative breeding flocks. Some preliminary studies have been conducted on this topic. He *et al.* [9] performed real-time, fluorescence-based quantitative PCR to detect the genomic DNA of *S. enteritidis* in the blood and internal organs of chicken after oral challenge at different time points, and they reported that the liver and spleen are the primary sites for *S. Enteritidis*. Further, Zeng *et al.* [10] analyzed the distribution of *S. Enteritidis* in internal organs in newly hatched chicken after oral challenge, found that all of the organs tested were positive at 12 h post-inoculation (PI), and the highest copy numbers of *S. enteritidis* in all tissue were heart and liver. Haider *et al.* [11] assessed gross tissue changes and clinical signs of PD in chickens PI, reporting that from blood, bacteria are seeded into the cells and tissues of different organs and also in different parts of the reproductive tract; moreover, the infection persists in ovary and egg follicles and transmits into laid eggs and then to hatched chicks.

On farms, the traditional method to detect *S. pullorum* infection mainly involves the collection of feces and sera to test for antigens and antibodies, respectively, but it remains unclear whether this method is reliable in cases of substantial infection [12]. Only a few studies have explored the organ dissemination pattern of *S. pullorum* in chickens, particularly in young chickens, and even fewer have assessed *S. pullorum* shedding and antibody production regularity in chickens of different ages PI. Therefore, herein we aimed to characterize the dissemination pattern in different organs PI with *S. pullorum*, to explore the regularity *S. pullorum* shedding in the faeces, and risk of horizontal transmission in the environment, and to investigate antibody production regularity in *S. pullorum*-infected chickens. We believe that our findings should facilitate the screening of *S. pullorum*-negative chickens on farms.

## Results

### *S. pullorum* dissemination in different organs and shedding regularity

**Fig. 1:**
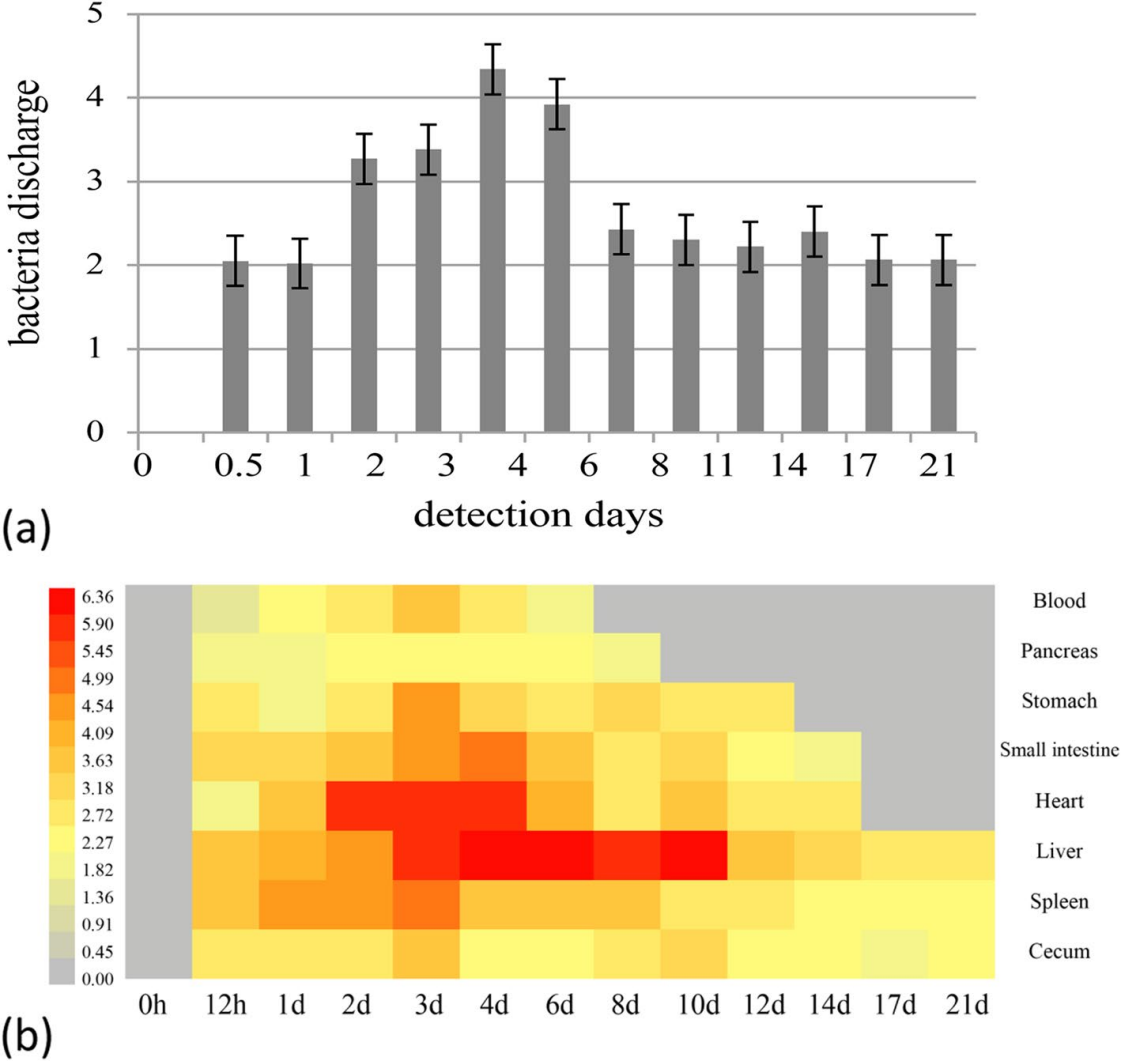
Regularity of (**a**) *S. pullorum* shedding and (**b**) dissemination in different organs, as detected using qPCR. Log10 copies of DNA target sequences per 0.2 mL blood, 0.5 g cecal contents and feces, and 30 mg of the liver, spleen, heart, stomach, small intestine, and pancreas

*S. pullorum* shedding in the faeces and organ dissemination pattern were quantitatively detected with qPCR. Figure 1a depicts the fecal shedding pattern of *S. pullorum* throughout the experiment. *S. pullorum* copy number in feces rose from zero to 1.07 × 10^2^ at 12 h PI, and then it quickly increased to the highest value (2.1 × 10^4^) at 4 days PI. The copy number then declined, decreasing to 2.6 × 10^2^ at 8 days PI. It stabilized within the next 13 days, with the copy number being 1.1 × 10^2^ on the last day (i.e., at 21 days PI).

Figure 1b shows the dissemination pattern of *S. pullorum* in different organs and at different time points. We found that the organs infected with the highest copy number of *S. pullorum* were the liver and heart. After 12 h, all organs tested in this study were infected by *S. pullorum*—the liver, spleen, and cecum carried *S. pullorum* throughout the experiment duration, while other organs, such as the pancreas, small intestine, and heart, did not carry *S. pullorum* during the last few days of the experiment. The most rapid clearing of *S. pullorum* was observed in the blood on 8 days PI, followed by the pancreas on 10 days PI, the stomach on 14 days PI, and finally the small intestine and heart on 17 days PI.

The liver showed the highest copy number of *S. pullorum* with approximately 1.7 × 10^6^ to 6.4 × 10^6^ copies of DNA target sequences/30 mg during 3–10 days PI, followed by the heart (7.9 × 10^5^ to 1.9 × 10^6^ copies of DNA target sequences/30 mg during 2–4 days PI), spleen (3.8 × 10^4^ to 1.4 × 10^5^ copies of DNA target sequences/30 mg during 1–3 days PI), small intestine (4.8 × 10^3^ to 1.0 × 10^5^ copies of DNA target sequences/30 mg during 2–6 days PI), and stomach (1.3 × 10^3^ to 3.5 × 10^4^ copies of DNA target sequences/30 mg during 2–4 days PI). Although the copy number of *S. pullorum* in the cecum was low (with the highest value being 1.0 × 10^4^ copies of DNA target sequences/0.5 g during 3–10 days PI), it was detected throughout the experiment duration.

### *S. pullorum* shedding and antibody production regularity in 10-day-old chickens

**Fig. 2:**
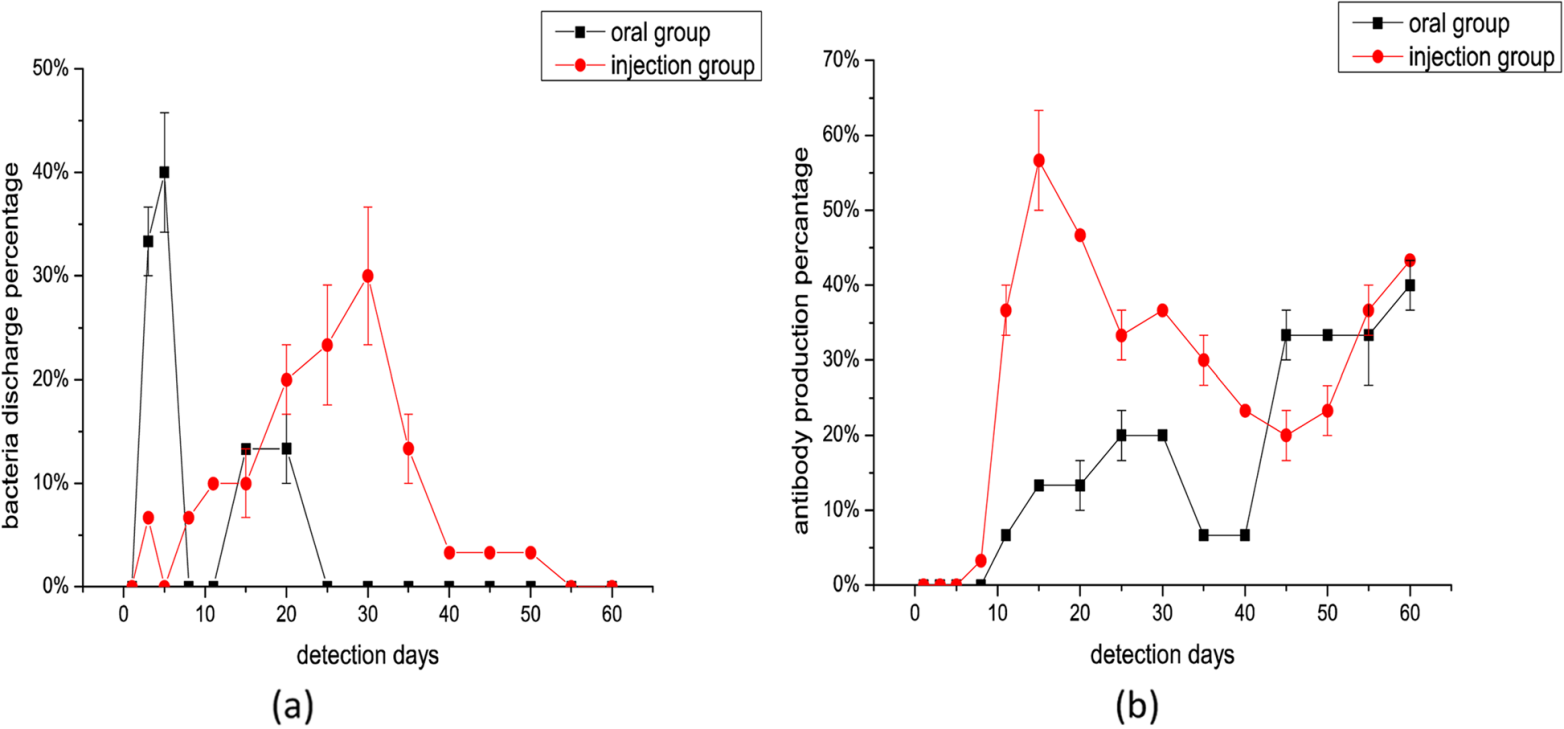
Regularity of (**a**) *S*. *pullorum* shedding and (**b**) antibody production in 10-day-old infected chickens

*S. pullorum* shedding (cloacal swabs) and antibody production (sera) were examined as described above, and the percentage of chickens that tested positive was calculated. As evident from Fig. 2, *S. pullorum* shedding and antibody production rates markedly fluctuated during the entire experiment (0%–40% and 0%–56.6%, respectively); the percentage of birds showing antibody production tended to increase, while that of those showing *S. pullorum* shedding tended to decrease.

*S. pullorum* shedding results can be summarized as follows: in the oral inoculation group, *S. pullorum* shedding appeared on the 3 days PI (33.3%); subsequently, the percentage of birds showing *S. pullorum* shedding rapidly increased, reaching the highest value (40%) on 5 days PI. After this, the percentage sharply dropped to zero, and *S. pullorum* was detected only on the 15 (13.3%) and 20 (13.3%) days PI. The intraperitoneal injection group showed *S. pullorum* shedding on 3 days PI (6.6%), followed by a short pause on 5 days PI. After 5 days, the percentage of birds showing *S. pullorum* shedding gradually increased, reaching the highest value on 30 days PI (30%), and it eventually became zero after 55 days PI.

With regard to serum antibody production, on 11 days PI with oral inoculation, serum antibodies were detected in the flock (6.6%). The percentage of antibody-positive birds first increased from 6.6% on 11 days to 20% on 30 days PI, and then after decreasing to 6.6% on 40 days PI, it again increased to 33.3% on the 55 days PI (“S”-shaped curve); the highest value of 40% was recorded on the 60 days PI. In the intraperitoneal injection group, serum antibody production was detected at 8 days PI (3.3%); the percentage of antibody-positive birds first increased (from 3.3% at 8 days to 56.6% at 15 days PI), then decreased (23.3% at 45 days PI), and then again increased to 43.3% at 60 days PI. *S. pullorum* shedding or antibody production was not observed at all in the negative control group.

On comparing *S. pullorum* shedding and antibody production between the oral inoculation and intraperitoneal injection groups, we found that the latter showed a longer duration of *S. pullorum* shedding; in fact, *S. pullorum* shedding could be detected during almost the entire experiment in the intraperitoneal injection group. By the end of the experiment, although there was a marked difference between the groups, the percentage of birds showing *S. pullorum* shedding and serum antibody production tended to be consistent in the two groups (0% for *S. pullorum* shedding and 40% for antibody production).

### *S. pullorum* shedding and antibody production regularity in 42-week-old chickens

**Fig. 3:**
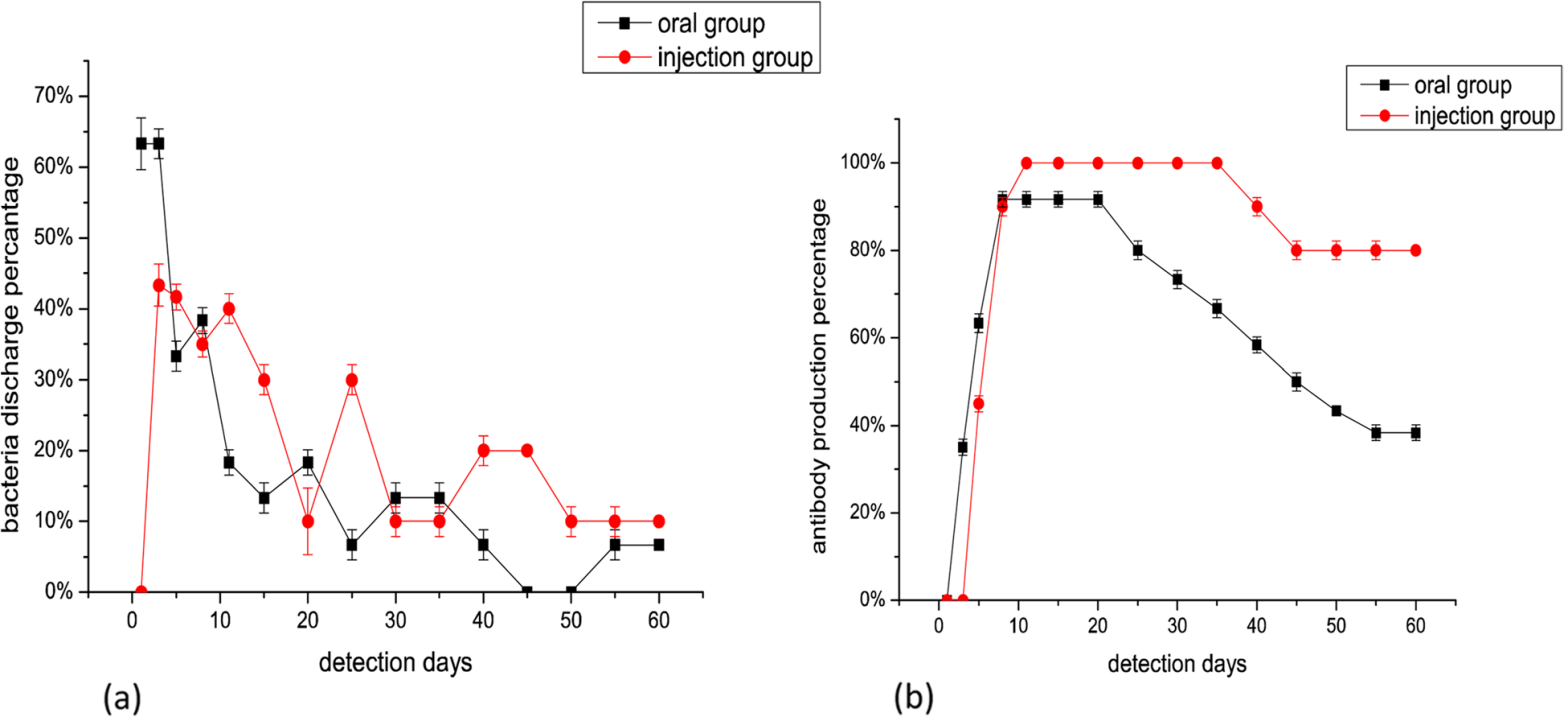
Regularity of (**a**) *S. pullorum* shedding and (**b**) antibody production in 42-week-old infected chickens

Figure 3a shows the trend of *S. pullorum* shedding. Although the curves appear variable to some extent, the overall trend is downward. On the first day PI, 63.33% birds in the oral inoculation group showed *S. pullorum* shedding, which was the highest during the entire experiment. This percentage dropped to zero on 45 days PI, and then slowly increased to 6.66% on 60 days PI. The overall trend in the intraperitoneal injection group was also downward. The difference between the groups was that the percentage of birds showing *S. pullorum* shedding in the oral inoculation group reduced to zero, but it remained steady at 10% in the intraperitoneal injection group. Further, in the intraperitoneal injection group, *S. pullorum* shedding was observed on 3 days PI (43.3%), and at 20, 30–35, and 50–60 days PI, the percentage of chickens showing *S. pullorum* shedding was approximately 10%, which was the lowest recorded value.

Figure 3b shows the regularity of antibody production. In the oral inoculation group, the percentage of antibody-positive chickens first rapidly increased and then gradually decreased. Antibody production was noted on 3 days PI (35%); the percentage of antibody-positive birds continued to increase from 3 days PI to 8 days PI, reaching the highest value of 91.67%. After 20 days PI, the percentage showed a sharp decline to 38.33% at 60 days PI. The intraperitoneal injection group showed the same the overall trend. The percentage of antibody-positive birds in the intraperitoneal injection group increased from 45% at 5 days PI to 100% at 11 days PI, remaining constant at 100% till 35 days PI. The percentage finally reduced to 80% at 45 days PI and remained constant at this value till the end of the experiment. It is notable that the decrease was more rapid in the oral inoculation group. The negative control group showed no *S. pullorum* shedding or antibody production at all.

To summarize, in comparison with the oral inoculation group, *S. pullorum* shedding and antibody production appeared later in the intraperitoneal injection group.

### Cohabitation experiment

**Fig. 4:**
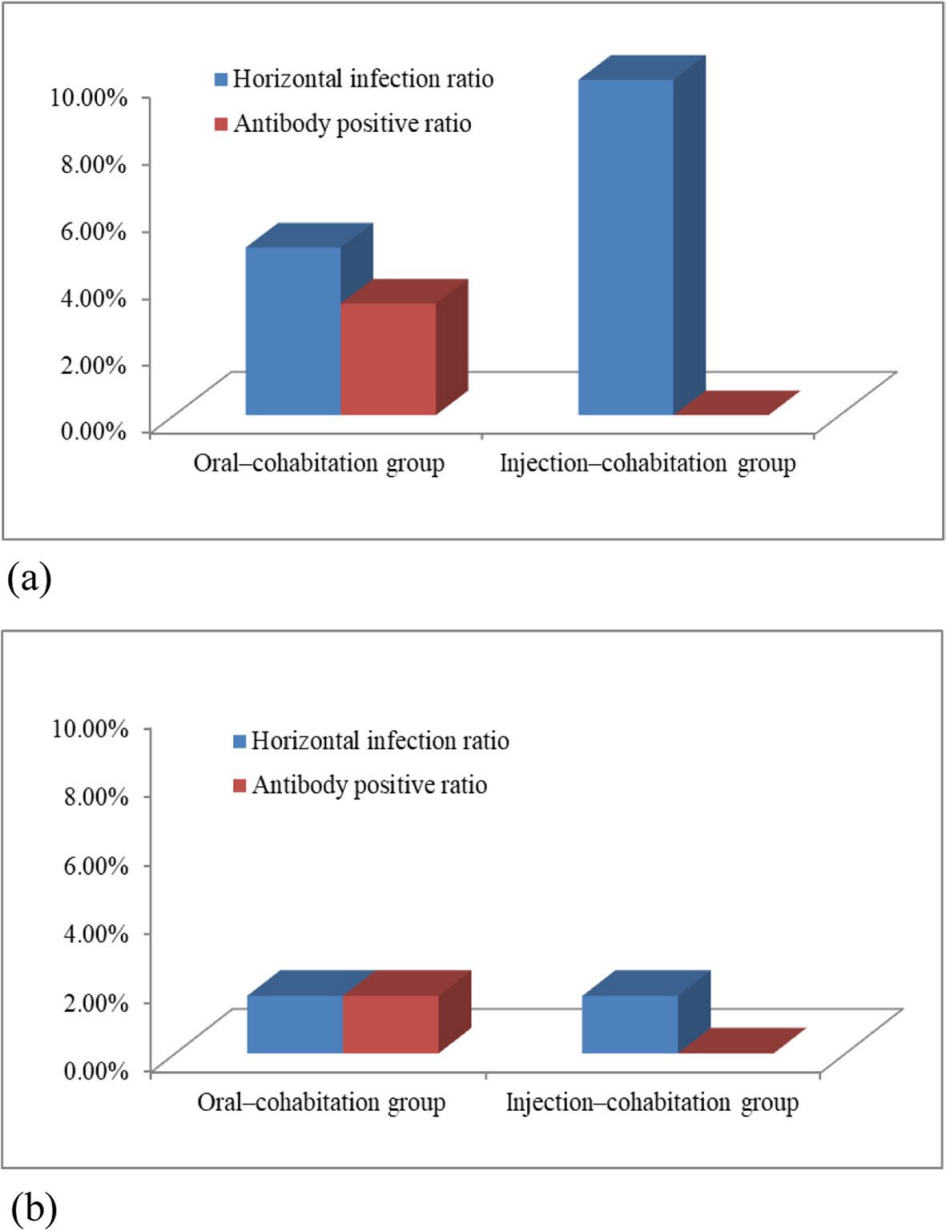
The horizontal infection ratio and antbody positive ratio of (**a**) 10-day-old chickens and (**b**) 42-week-old chickens in cohabitation experiment

Figure 4 depicts our cohabitation experiment results. According to *S. pullorum* shedding results, nine (15%) 10-day-old chickens in total were infected via the horizontal transmission of *S. pullorum* in cohabitation experiment: three (two at 3 days PI and one at 5 days PI) in the oral inoculation–cohabitation group and six (one each at 15, 20, 30, and 35 days PI and two at 11 days PI) in the intraperitoneal injection–cohabitation group. Further, according to antibody production results, the antibody positive ratio of 10-day-old chickens was 3.33% in cohabitation experiment (one chicken at 30 days PI and one at 50 days PI) in the intraperitoneal injection–cohabitation group. No antibody-positive chickens were found in the oral inoculation–cohabitation group (Fig 4a).

In 42-week-old chickens (Fig 4b), 3.33% (*n* = 2) chickens showed *S. pullorum* shedding, which was detected at 8 and 11 days PI in the oral inoculation–cohabitation and intraperitoneal injection–cohabitation groups, respectively. Only 1 chicken (1.67%) showed antibody production at 15 days PI in the intraperitoneal injection–cohabitation group.

**Fig. 5:**
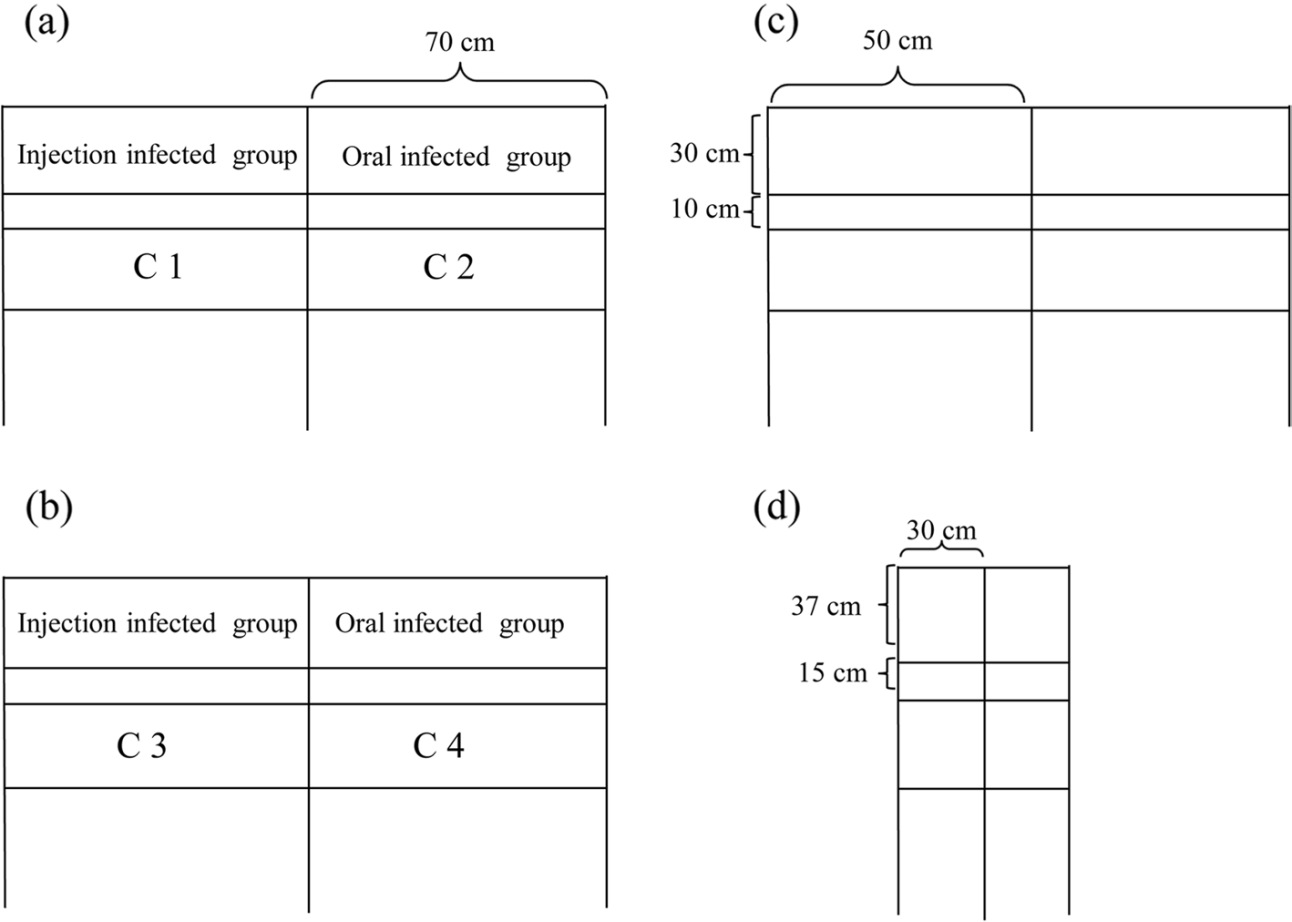
Groups C1 and C2 = 10-day-old chickens (**a**); groups C3 and C4 = 42-week-old chickens (**b**). (**a**) and (**b**) are the front view of cages; (**c**) and (**d**) is the vertical view of cages

The structure of the chicken cage in the cohabitation test and the position of the cohabiting *S. pullorum*-free chickens are shown in Fig 5a-d. Overall, we found that regardless of the infection method or location of the flock, *S. pullorum* was horizontally transmitted. From the perspective of the structure of the chicken cage, the distance between *S. pullorum* infected and *S. pullorum*-free chickens is very close, only 10-15cm, which cannot prevent the spread of pathogens. Our data not only demonstrate the horizontal transmission ability of *S. Pullorum,* but also proves the degree of infection in the cohabitation environment. Among different ages of *S. pullorum*-negative chickens, comparison with 42-week-old chickens, 10-day-old chickens showed are more susceptible to *S. pullorum* in the cohabitation environment.

## Discussion

Upon establishing an infection, *S. pullorum* can evade host immune defense and parasitize both the spleen and reproductive tract for >40 weeks before being transmitted via the digestive or reproductive tract. The infection cycle includes three stages: (I) invading the intestine and parasitizing the gastrointestinal tract epithelium [13], (II) invasion of macrophages and dendritic cells and establishment of systemic infection in different tissues via the lymphatic system, which is crucial for long-time persistence [14], and (III) development of the infection outcome, which can be either clearance, death, or carrier state [15]. Herein chickens of different ages were artificially infected with *S. pullorum* using different inoculation routes, and *S. pullorum* organ dissemination and shedding patterns, serum antibody production, and horizontal transmission risk were determined.

Young birds are very susceptible to *S. pullorum* infection and their underdeveloped immune systems encounter difficulties in tackling bacterial invasion. On exploring the organ dissemination pattern of *S. pullorum* in 2-day-old chickens, we found that the liver, spleen, and cecum were mainly infected. Our data support the concept that the liver and spleen are the primary lymphoid tissues where bacteria localize and multiply [7] and which exhibit the most severe histopathological lesions (16). The heart was as affected as the liver; in fact, both the organs showed the highest levels of infection in this study, which is consistent with the results of a previous study [16].

On farms, the most common methods to detect *S. pullorum* infection include determining the presence of bacteria in feces and assessing the levels of serum antibodies [17]. Thus, elucidating the regularity of *S. pullorum* shedding and antibody production, which seem to rely on the maturity of the immune system and its ability to clear bacteria, in chickens of different ages is pivotal. We observed that 42-week-old chickens showed higher *S. pullorum* shedding than 10-day-old chickens. This could be because at the sexually mature stage, *S. pullorum* population must have rapidly increased in the reproductive tract of 42-week-old chickens owing of their increased susceptibility to *Salmonella* [18]. With regard to serum antibody production, the percentage of antibody-positive 10-day-old chickens first increased, then decreased, and then again increased (“S”-shaped curve), while that of antibody-positive 42-week-old chickens slightly decreased during the later stages of the experiment. Serum antibody production in 10-day-old chickens was lower and more unstable. Therefore, when using the plate agglutination test to detect serum antibodies for PD, the age of chickens should not be too low to avoid deviations in test results.

In this study, we used oral inoculation for *S. pullorum* challenged in different ages chickens. Many studies have challenged chickens with *Salmonella* by oral inoculation, which is consistent with our inoculation method, and may be similar to natural infection way in farm environment [9, 10]. However, Nazir et al. believed that only a small proportion of the *S. Gallinarum* in oral inoculation were able to reach visceral organs due to the antagonistic effects of low gastric pH and inhibitory effects of the normal intestinal flora, and may not cause systemic infection. Compared to oral inoculation, the intraperitoneal route of infection could be an alternative to overcome these difficulties in experimental trials [19]. Although more severe and observed at earlier stages of infection and in more birds in intraperitoneal inoculation chickens, there was no significant difference between oral and intraperitoneal inoculation *S. Gallinarum* infected chickens in the gross and histopathological changes of visceral organs, even the frequency of isolation from internal organs and fecal sheddings [19]. Similar results were obtained in the study of *Salmonella Zega* on the pathogenicity in chicks by Mshelbwala *et al.* [20, 21]. Based on the conclusions of previous studies, we believed that oral and intraperitoneal inoculation should be used in this experimental trial to comprehensively evaluate the regularity of fecal shedding and antibody production of *S. pullorum* in infected chickens.

In the current study, we found that in comparison with the intraperitoneal injection group, all chickens in the oral inoculation group, regardless of their age, showed *S. pullorum* shedding soon after the first day PI. This could be because the oral inoculation route directly introduces the bacteria in the digestive tract [22], whereas the intraperitoneal injection route requires the bacteria to first invade tissues and organs before entering the gastrointestinal tract. However, we observed that *S. pullorum* infection in the intraperitoneal injection group lasted longer, suggesting that this route of administration increased the number of bacterial cells in the blood and organs. In previous studies, the rate of antibody production in chickens injected with *Salmonella* was slower than in those that were orally inoculated, but antibody levels were higher and the response lasted for a longer duration [23, 24], which is consistent with our results. From an overall perspective, we believe that the route of inoculation plays a key role: oral inoculation induces a rapid response by the intestinal mucosal immune system, while inoculation by injection mainly induces humoral immunity.

Our data analyses show that there exists a correlation between *S. pullorum* shedding and antibody production. We believe that the production of serum antibodies can effectively reduce *S. pullorum* shedding by chickens through the gastrointestinal tract [25]. However, the appearance of serum antibodies cannot completely eliminate the infection because *S. pullorum* parasitizes cells, thereby impairing humoral immunity [26]. *S. pullorum* was thus detected in some infected chickens intermittently during the study even if serum antibody production was observed [15, 27]. In addition, we found that regardless of the age of chickens, neither antibody production nor *S. pullorum* shedding was noted in some cases. These data highlight that when detecting the presence of *S. pullorum* infection, some false-negative results should be expected; to obtain reliable results, multiple timespans and replicates should be assessed.

Transovarian infection resulting in the infection of the egg and subsequently of the progeny is one of the most important modes of *S. pullorum* transmission [28]. *S. pullorum* can, however, still horizontally spread when chickens come into contact with infected feces or pollutants [29]. Our cohabitation experiment results showed that the overall infection rate was low, which may be related to the host specificity of *S. Pullorum* [30]. We also found different levels of infection in different ages of chickens. In general, 10-day-old chickens showed higher *S. pullorum* shedding and antibody production than 42-week-old chickens; this could be attributed to the underdeveloped immune system of young birds, which makes them more susceptible to *S. pullorum* infection [31]. In addition, even environmental factors, such as stocking density and temperature, could have influenced our cohabitation experiment results. Under normal conditions, the stocking density of chicks and optimum environment temperature are higher than that of adult chickens, potentially resulting in a more closed housing conditions [32]. In addition, in large-scale farms, common stepped or stacked chicken cages cannot effectively prevent the horizontal spread of *S. pullorum* due to the close distance between chicken cages [33]. These environmental factors may increase the ability of *S. pullorum* to spread horizontally. The application of biosafety measures to block this horizontal transmission can play a positive role in *S. pullorum* infection prevention and control. Previous studies have reported that rats [17], wild and game birds, insects, and mammals can be a constant reservoir of *S. pullorum* and that they may play a key role in its spread on farms [29]. The detection and elimination of infected chickens is an effective measure to prevent the spread of *S. pullorum* infection—verifying the source of chickens and maintaining hygiene can facilitate *S. pullorum* elimination from farms [17].

## Conclusion

After artificial inoculation of *S. pullorum*, the main organs infected of chickens are liver, heart, and spleen. The changes in *S. pullorum* shedding and serum antibody production in different age chickens showed clear regularity. The production of serum antibodies can reduce the ratio of bacteria in the digestive tract. The results of cohabitation experiment group indicate that the direct horizontal transmission ability of *S. pullorum* among chickens. we believe that our findings provide insights into how to screen *S. pullorum*-negative chickens on livestock farms; moreover, the data reported herein should help in the development of methods to prevent and eradicate *S. pullorum*.

## Methods

### Bacterial strain, growth conditions, and inoculum preparation

The standard strain *S. pullorum* ATCC 13036 was obtained from the China Institute of Veterinary Drug Control. The strain was revived on xylose lysine deoxycholate (XLD) medium (Beijing Laboratory Biology Technology Co., Ltd) and cultured at 37°C for 24 h. Then, typical colonies were selected from the XLD agar and transferred to the buffered peptone water (BPW; Hopebiol, Qingdao, China) and cultured at 37°C for 12 hours. Subsequently, the cultured bacterial suspension was inoculated on sheep blood agar plate, and cultured at 37°C overnight, the lawn was washed with phosphate buffered saline, and The concentration of ATCC 13036 suspension was determined by the plate dilution method. We determined the median lethal dose (LD50) of ATCC 13036 by pre-experiment, and the determination results are shown in Table S1. The 7-day LD50 of ATCC 13036 for 2-day-old chicks was 1.835 × 10^9^ cfu, with a 95% confidence interval of 6.039 × 10^8^ cfu and 9.163 × 10^9^ cfu. The concentration of ATCC 13036 suspension, intended to be used as the inoculum, was adjusted to 1.0 × 10^8^ (for 2- and 10-day-old chickens) and 1.0 × 10^9^ (for 42-week-old chickens) cells per chicken.

### *S. pullorum* dissemination and shedding patterns PI

To evaluate *S. pullorum* shedding and organ dissemination pattern PI, we used 72 specific-pathogen-free 2-day-old breeding chickens, which were purchased from Beijing Merial Vital Laboratory Animal Technology Co., Ltd. They were infected with 0.2 mL *S. pullorum* (1.0 × 10^8^ cells/chicken) using the oral inoculation route; negative controls were administered the same amount of sterile saline and kept isolated from the test environment. The feeding method was based on that reported by a previous study [34]. All feed and drinking water were bacteria-free. The environment and housing facilities met the guidelines put forth by the National Standards of Laboratory Animal Requirements of Environment and Housing Facilities of China : temperature was maintained at 20°C–26°C and humidity at 40%–70%. A 10:14 light–dark cycle was applied.

At 0, 0.5, 1, 2, 3, 4, 6, 8, 11, 14, 17, and 21 days PI, six chickens were randomly selected from each group and euthanasia was performed by sedation using a Rompun/Ketamine (1 mg/kg) mixture as an intramuscular thigh injection followed by an intravenous wings injection of Pentobarbitone (150 mg/kg), and for testing purposes, 0.2 mL blood; 30 mg of the liver, spleen, heart, stomach, small intestine, and pancreas; and 0.5 g cecal contents and feces were collected. All samples were stored in the dark at 0°C, and DNA was extracted from them within 30 min using the Tiangen DNA kit [35] (Tiangen Biotech, Beijing, China), according to manufacturer instructions. qPCR was performed to quantitatively detect *S. pullorum* in all samples, as previously described [36]. Briefly, a 155-bp region of *INVA* was amplified (forward primer: 5′-CCCGCTGCCGGTATTTGTTA-3′; reverse primer: 5′-TCAGTCCTAACGACGACCCT-3′), which is a unique gene in *Salmonella* and shows good specificity. The total reaction volume was 20 μL, and qPCR was performed with a SYBR Green qPCR kit (GeneCopoeia, China). The standard curve, CT values, and amplification efficiencies were analyzed using BIO-RAD iQ5 software, and each sample contains 3 replicate assays.

### *S. pullorum* shedding and antibody production regularity in 10-day-old and 42-week-old chickens PI

Ninety 10-day-old chickens and 180 42-week-old laying-stage hens were purchased from Anhui Bocheng Agriculture and Animal Husbandry Technology Co., Ltd. (China), and randomly divided into three groups: oral inoculation, intraperitoneal injection, and control groups. All birds were *S. pullorum* negative, as assessed using the serum plate agglutination test [37] and the isolation test [38]. The ‘Animal Research: Reporting in-Vivo Experiments’ (ARRIVE) [39] has been considered and integrated into this study protocol were applicable.

ATCC 13036 concentration was adjusted as described above (1.0 × 10^8^ cells for 10-day-old chickens and 1.0 × 10^9^ cells for 42-week-old chickens) in a total volume of 0.5 mL to inoculate 10-day-old and 42-week-old chickens via the oral and intraperitoneal routes, respectively. The control group was inoculated with the same amount of sterile 0.85% saline, and birds were kept in a different room to isolate them from the test environment. Cloacal swabs and sera were collected from all chickens on the 1, 3, 5, 8, 11, 15, 20, 25, 30, 35, 40, 45, 50, 55 and 60 days PI. *S. pullorum* shedding in the faeces was then examined. Three repetitions of sampling and detection were carried out at each time point, and the percentage of *S. pullorum* Ag (cloaca swabs) and Ab (sera) positive chickens was the average of three detection results. *S. pullorum* was isolated from the cloacal swabs according to the “*Salmonella* detection method for animal and animal products” (NY/T550-2002, in Chinese). Briefly, the sample was enriched with buffered peptone water, Rappaport–Vassiliadis medium, and selenite cystine broth medium, and then streaked on xylose lysine deoxycholate, bismuth sulfite, and Hektoen enteric agar. *S. pullorum* colonies appeared with black centers on the aforementioned media, with the bacteria being Gram negative. The biochemical test results were as follows: the slope layer of the TSI reaction produced alkali, while the bottom layer was acid genic; cells were positive for ornithine decarboxylase and lysine decarboxylase and negative for urease and galactitol. The dynamic test results were negative too. The identity of any “suspicious” colonies was determined using the *Salmonella pullorum* Ag Test Kit (cloaca swabs). The detection and determination of serum antibodies against PD were carried out in accordance with the “quarantine protocol for fowl typhoid and pullorum disease” (SN/T1222-2012) and using the *Salmonella pullorum* Ab Test Kit (sera). Chickens were euthanized by CO_2_ inhalation when they were reached a predetermined humane endpoint.

### Cohabitation experiment

Sixty 10-day-old and 60 42-week-old chickens were used for cohabitation experiments to test the horizontal transmission ability of *S. pullorum* [22]. For this experiment, all chickens were inoculated with 0.5 mL sterile 0.85% saline (i.e., the cohabitation group), and to test whether different methods of inoculation have an impact on the ability of *S. pullorum* to spread, they were kept in different cages in the same room with the oral inoculation and intraperitoneal injection groups (Fig. 5a–d). All birds were *S. pullorum* negative, as assessed using the serum plate agglutination test [37] and the isolation test [38].

At 1, 3, 5, 8, 11, 15, 20, 25, 30, 35, 40, 45, 50, 55, and 60 days PI, cloacal swabs and sera were collected from each cohabitation group for further analyses, such as Gram staining, biochemical testing, and *S. pullorum* Ag (cloaca swabs) and Ab (sera) testing, which were performed using the same methods as described above.

### Statistical analysis

Data were preliminarily processed using excel 2010 (Microsoft Office, WA, USA), the further statistical analysis was performed by SPSS v16.0 (SPSS Inc., Chicago, IL, USA ), and the Graphpad prism v7 software (GraphPad Software Inc., San Diego, CA, USA) was used to make the figures. The values are presented as mean ± SD.

## Supplementary Information

**Additional file 1: Table S1.** The lethality assay of *S. pullorum* ATCC 13036 for 2-day-old chickens.

## Data Availability

The datasets used and/or analysed during the current study are available from the corresponding author on reasonable request.

## Abbreviations

*S. Pullorum*: *Salmonella enterica serovar Gallinarum biovar Pullorum*
Ag: Antigen
Ab: Antibody
PI: Post infection
PD: Pullorum disease
TSI: Triple-sugar-iron-agar medium
qPCR: Quantitative real-time polymerase chain reaction
BPW: Buffered peptone water
XLD: Xylose lysine deoxycholate
