# Supplementary material for: Evaluating Salmonella pullorum dissemination and shedding patterns and antibody production in infected chickens
Source: BMC Vet Res. 2022 Jun 24;18:240. doi: 10.1186/s12917-022-03335-z (PMC9229423; doi:10.1186/s12917-022-03335-z)
Supplement: Supplementary file 1 — Additional file 1: Table S1. The lethality assay of S. pullorum ATCC 13036 for 2-day-old chickens. [file 12917_2022_3335_MOESM1_ESM.docx]

Table S1. The lethality assay of *S. pullorum* ATCC 13036 for 2-day-old chickens

| Dose (CFU) | Number of embryos found dead at indicated day post-inoculation | | | | | | | Accumulated chicken mortality (%) |
| --- | --- | --- | --- | --- | --- | --- | --- | --- |
|  | 1 | 2 | 3 | 4 | 5 | 6 | 7 |  |
| 8.7 × 10^9^ | 1 | 2 | 3 | 1 | 1 | 0 | 0 | 80 (8/10) |
| 8.7 × 10^8^ | 0 | 1 | 1 | 0 | 1 | 0 | 0 | 30 (3/10) |
| 8.7 × 10^7^ | 0 | 0 | 0 | 0 | 1 | 0 | 0 | 10(1/10) |
| 8.7 × 10^6^ | 0 | 0 | 0 | 0 | 0 | 0 | 0 | 0(0/10) |
| 8.7 × 10^5^ | 0 | 0 | 0 | 0 | 0 | 0 | 0 | 0(0/10) |
| PBS | 0 | 0 | 0 | 0 | 0 | 0 | 0 | 0(0/10) |
